# A scalable, virtual weight management program tailored for adults with type 2 diabetes: effects on glycemic control

**DOI:** 10.1038/s41387-023-00234-6

**Published:** 2023-04-06

**Authors:** John W. Apolzan, Jessica Gokee LaRose, Stephen D. Anton, Robbie A. Beyl, Frank L. Greenway, Edmond P. Wickham, Autumn Lanoye, Melissa N. Harris, Corby K. Martin, Tiffany Bullard, Gary D. Foster, Michelle I. Cardel

**Affiliations:** 1 Pennington Biomedical Research Center, Baton Rouge, LA USA; 2 Virginia Commonwealth University School of Medicine, Richmond, VA USA; 3 University of Florida, Gainesville, FL USA; 4 WW International, Inc., New York, NY USA; 5 Center for Weight and Eating Disorders, Perelman School of Medicine, University of Pennsylvania, Philadelphia, PA USA; 6 Department of Health Outcomes & Biomedical Informatics, University of Florida College of Medicine, Gainesville, FL USA; 7 Center for Integrative Cardiovascular and Metabolic Disease, University of Florida, Gainesville, FL USA

**Keywords:** Type 2 diabetes, Obesity

## Abstract

**Background:**

The objective was to test the efficacy of a scalable, virtually delivered, diabetes-tailored weight management program on glycemic control in adults with type 2 diabetes (T2D).

**Methods:**

This was a single arm, three-site clinical trial. Participants had baseline HbA1c between 7–11% and BMI between 27–50 kg/m^2^. Primary outcome was change in HbA1c at 24 weeks. Secondary outcomes were changes in body weight, waist circumference, the Diabetes Distress Scale (DDS), quality of life (IWQOL-L), and hunger (VAS). Generalized linear effects models were used for statistical analysis.

**Results:**

Participants (*n* = 136) were 56.8 ± 0.8 y (Mean ± SEM), 36.9 ± 0.5 kg/m^2^, 80.2% female, 62.2% non-Hispanic white. Baseline HbA1c, weight, and total DDS score were 8.0 ± 0.09%, 101.10 ± 1.47 kg, and 2.35 ± 0.08, respectively. At week 24, HbA1c, body weight, and total DDS decreased by 0.75 ± 0.11%, 5.74 ± 0.50%, 0.33 ± 0.10 units, respectively (all *p* < 0.001). Also, at week 24, quality of life increased by 9.0 ± 1.2 units and hunger decreased by 14.3 ± 2.4 units, (both *p* < 0.0001).

**Conclusions:**

The scalable, virtually delivered T2D-tailored weight management program had favorable and clinically meaningful effects on glycemic control, body weight, and psychosocial outcomes.

## Introduction

Diabetes is a debilitating, deadly, and costly disease [1, 2]. In 2012, the cost of care for diabetes totaled $245 billion, including $176 billion in direct medical costs, which is 2.3 times higher than costs in people without diabetes [3].

The American Diabetes Association (ADA) Standards of Care underscore the multiple benefits of weight management in the effective management of type 2 diabetes (T2D) [4]. Among those with T2D, the LookAHEAD study demonstrated that an intensive lifestyle intervention produced a 7.9% greater reduction in weight and a 0.5% greater reduction in hemoglobin A1c (HbA1c) than did a usual care approach at 1 year [5].

While clinic-based lifestyle interventions that reduce both weight and HbA1c are the most studied [6], their high cost and finite number, limit reach, accessibility, and impact for a large number of patients [7]. Community-based weight management programs are more affordable [8] and accessible than clinic-based modalities [9], and have been shown to be effective in promoting weight loss and improvements in glycemic control in adults [10] A randomized trial of a modified WeightWatchers (WW) program for patients with T2D showed improved glycemic control (HbA1c) and significant reductions in weight compared to standard of care diabetes nutrition counseling and education [11]. Here, we test the efficacy of a new WW program tailored for individuals with T2D and delivered virtually. We hypothesized that the program would result in clinically meaningful reductions in HbA1c [6].

## Methods

This single-arm, three-site trial included Pennington Biomedical Research Center in Baton Rouge, LA, University of Florida in Gainesville, FL, and Virginia Commonwealth University in Richmond, VA. All participants were given verbal and written explanations about the study, provided written informed consent, and received incentives for data collection visits ($50 at 0 and 12 weeks, $125 at 24 weeks). Participants were recruited in cohorts, ranging from 7 to 31 participants (mean *n* = 17), between April–June 2021. The study was approved by the Institutional Review Boards at all three sites and registered at ClinicalTrials.gov (NCT04804774).

Participants had a reported diagnosis of T2D, were 18–70 y, objectively measured HbA1c between 7–11% and Body Mass Index (BMI) 27–50 kg/m^2^, on a stable medication regime for >3 months, and under a physician’s care for the management of T2D. The average length of time on T2D medication was 4 years. The number of participants on diabetes medication classes are as follows: (1) Rapid – Acting Insulin, *n* = 14; (2) Intermediate – Acting Insulin, *n* = 2; (3) Long – Acting Insulin, *n* = 21; (4) Combination Insulin, *n* = 1; (5) Biguanides, *n* = 40; (6) Sulfonylureas, *n* = 16; (7) Glucagon-like Peptide-1 agonists, *n* = 27; (8)Thiazolidinediones, *n* = 1; (9) Meglitinides *n* = 1; (10) DPP-4 Inhibitors, *n* = 11, (11) SGLT-2 Inhibitors, *n* = 16; and (12) Dopamine agonists, *n* = 1. Also, full exclusion criteria are listed below:

### Exclusion criteria

1. Participation in a weight control program within the past 3 months
2. Weight loss of ≥5 kg in the previous 6 months
3. Taking prescription or OTC weight loss medications within last 4 weeks
4. History of a surgical procedure for weight loss at any time (e.g. gastroplasty, gastric by-pass, gastrectomy or partial gastrectomy, adjustable banding, gastric sleeve)
5. History of major surgery within three months of enrollment
6. Type 1 diabetes
7. Renal insufficiency consisting of potassium over 5.5 (mmol/L) on a non-hemolyzed specimen, or a creatinine over 2.5 mg/dL
8. Bilirubin over 3 (mg/dL) or an albumin less than 3 (g/dL)
9. ALT > 3 (IU/L) times the upper limit of normal (normal range is 7–56)
10. Evidence of more than 1 severe hypoglycemic event (episode requiring emergency medical services) in the past 12 months, unless the participant’s treating physician provides written clearance for participation
11. Hemoglobinopathy that interferes with measurement of hemoglobin A1c
12. Those on higher doses of diuretics (furosemide 40 mg or higher or comparable)
13. Unstable heart disease (an ongoing workup or treatment for a cardiac symptom such as unstable angina, coronary ischemia)
14. Presence of implanted cardiac defibrillator
15. Blood pressure ≥180/100 mm Hg. If a potential participant has a BP above the inclusion criteria it is acceptable to re-test this potential participant within one week of the original test
16. Thyroid disease for which the participant is untreated or has had treatment changed within the last 6 months. History of thyroid disease or current thyroid disease treated with a stable medication regimen for at least 6 months is acceptable
17. Orthopedic limitations that would interfere with ability to engage in regular physical activity
18. Uncontrolled gastrointestinal disorders including chronic malabsorptive conditions, peptic ulcer disease, Crohn’s disease, chronic diarrhea or active gallbladder disease
19. Current cancer or cancer treatment, or a history of cancer or cancer treatment within the last 3 years. Persons with successfully resected non-melanoma carcinoma of the skin may be enrolled
20. Dementia, psychiatric illness, or substance abuse that may interfere with adherence (e.g. illness that is currently unstable or resistant to first-line therapy; substance abuse in the past year)
21. History of clinically diagnosed eating disorders including anorexia nervosa or bulimia nervosa
22. Women who are pregnant, lactating, trying to become pregnant or unwilling to use an effective means of birth control
23. Currently consuming >14 alcoholic drinks (1 drink = 12 fl oz beer, 4 fl oz wine or 1.5 fl oz liquor) per week and unwilling to limit intake to less than 3 drinks per drinking day during study participation
24. Participation in another clinical trial within 30 days prior to enrollment
25. Any other condition or factor which in the opinion of the study physician or investigator makes it inadvisable for the candidate to participate in the trial

As noted, specific drug exclusion criteria are as follows: (1) Anti-obesity medications (prescription or OTC weight loss medications) in the last 4 weeks including bupropion-naltrexone, liraglutide, phentermine, phentermine-topiramate, and orlistat. (GLP-1 Antagonists were acceptable in the lower dose for diabetics (if stable dosage for >3 months) but not at the higher dose which is FDA-approved for weight loss. (Tirzepatide was not FDA approved until May 2022, so no participants were taking the medication.) and (2) Diuretics with a dose that exceeds 40 mg (furosemide 40 mg or higher or comparable such as bumetanide, ethacrynic acid, and torsemide).

Participants completed 3 study visits including the screening/baseline visit and follow up visits at 12 (midpoint) and 24 weeks (post). All follow-up visits should have occurred within a ±7-day window but could occur within ±14 days. Participants were recruited in cohorts that ranged from 7 to 31 participants (mean *n* = 17) between April– June of 2021. Height, demographics, and medical history were assessed at screening/baseline. Key outcome measures included HbA1c, weight, waist circumference, blood pressure, diabetes distress (Diabetes Distress Scale; DDS) [12] and Impact of Weight on Quality of Life – Lite (IWQOL-L) [13], and hunger [14, 15].

For HbA1c, whole blood was collected via venipuncture. Trained research personnel measured height at baseline and body weight using a standardized digital weight scale, with participants wearing light clothing and shoes removed. Height was measured using a standardized height dynamometer. Waist circumference was measured in a horizontal plane around the abdomen at the level of the iliac crest. An average of the two closest measurements will be used for analyses. For height, weight, and waist circumference, measurement was performed twice, with a third measurement if the first 2 measurements deviate more than 0.5 cm or 0.5 kg.

The DDS is a measure of diabetes-related distress [12]. It consists of 17 items scored on a 1–6 scale, with higher scores indicating higher distress. The DDS comprises four subscales (emotional burden, physician-related distress, regimen-related distress, and interpersonal distress) and a total score. A total or subscale score > 2.0 (moderate distress) is considered clinically significant [16].

The IWQOL-L is a self-report measure of quality of life [13]. This measure is distinct from other measures of quality of life because it addresses this concept as it specifically relates to individuals with obesity. There are 31 items rated on a 1 (Never True) to 5 (Always True) point Likert scale with higher scores indicating more distress and a poorer quality of life. Five subscales are derived: physical function, self-esteem, sexual life, public distress, and work.

Retrospective Visual Analogue Scale (VAS) was used to measure average ratings of hunger that participants experienced over the past week. This method of collecting VAS data has been found to be consistent with daily assessments of satiety [15], and support has been found for the reliability and validity of VAS for measuring subjective states related to energy intake [14].

Following baseline assessments, eligible participants received the T2D-tailored WW program, which included access to weekly virtual workshops, weekly check-ins, the WW App, and a private online community. The program encouraged healthy habits with topics specific to T2D in the areas of food, activity, mindset, and sleep.

The intervention was delivered weekly via virtual group workshops. Each workshop lasted 30–60 min and included a new topic related to building healthy habits, behavioral skills to support behavior change, and group discussion. Through the WW app and website, participants were able to track their weights, dietary intake, physical activity; access progress reports; and complete weekly check-ins. The app also provided recipes, behavior change content, and T2D-specific information.

The core of the WW food program is the SmartPoints® system which assigns each food and beverage a SmartPoints® value per portion based on calories, protein, fiber, added sugar, saturated fat and unsaturated fat. In addition, foods that form the foundation of a healthy dietary pattern as recommended by the 2020–2025 Dietary Guidelines and global food-based dietary recommendations. (e.g., fruit, vegetables, whole grains, lean proteins) are assigned a SmartPoints® value of zero (ZeroPoint foods) and do not need to be measured or tracked. Based on glycemic control, foods higher in carbohydrates (e.g., fruit, yogurt, whole grains), are no longer ZeroPoints encouraging people with diabetes to weigh, measure, and track them. Participants were encouraged to focus on other zero point foods, such as lean proteins (e.g. skinless chicken and turkey breast), high fiber legumes (e.g. beans and peas), and healthy fats (e.g. avocado) that were lower in carbohydrates and/or higher in protein and less likely to impact blood sugar. Participants were provided with a personalized daily and weekly SmartPoints® budget which was designed to create an energy deficit of ~750 kcal/day using the Mifflin St Jeor equation [17].

### Statistical analyses

Analyses adhered to the intent-to-treat principle; missing data were accounted for using maximum likelihood estimation. General linear mixed effect models adjusted for sex were used to evaluate changes over time in HbA1c and secondary outcomes (including percent change) at baseline, 12, and 24 weeks. Results are presented as mean±standard errors or overall percentages. Testing of differences employed either *T*-tests, or Chi-squared tests for percentages. Planned exploratory analysis looked at HbA1c subgroups of >8% or ≤8% HbA1c.

### Power calculation

The sample size calculation was based on a previous WW study with T2D participants that found a 0.6 ± 1.4% decrease in HbA1c at 6 months [11]. To ensure adequate power, we anticipated a HbA1c reduction that was 20% less with a 20% higher standard deviation than previously found (0.5 ± 1.7%). The level of significance was 0.05 and was based on a two-sided one-sample *t*-test. Thus, a sample size of 120 subjects provided 90% power to detect this difference.

## Results

**Fig. 1:**
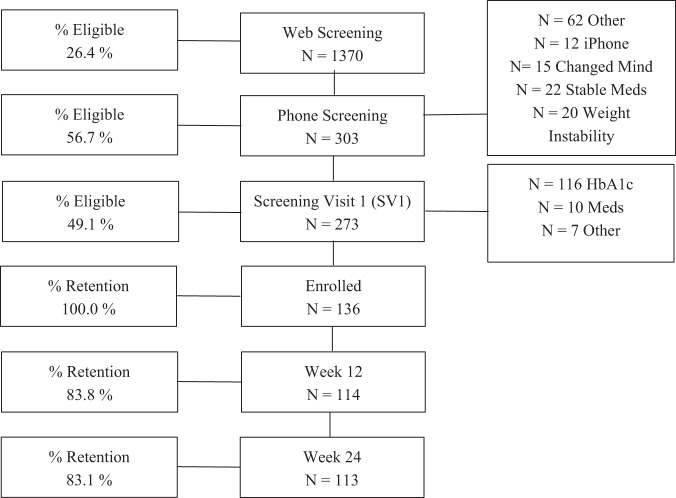
CONSORT Diagram. Not all persons performed Web Screening. Study was performed from April 2021 till December 2021 in Baton Rouge, Louisiana; Richmond, Virginia; and Gainesville, Florida.

The flow of participants from initial screening through week 24 is shown in Fig. 1. Baseline characteristics and changes at 12 and 24 weeks are described in Table 1. HbA1c significantly decreased (0.75 ± 0.11%) at week 24. Also, those with an initial HbA1c ≥ 8 (8.95 ± 0.12%, 99.65 ± 1.87 kg; *n* = 52), significantly reduced HbA1c by 1.35 ± 0.16 (*p* ≤ 0.0001) and body weight by −4.97 ± 0.58% (*p* ≤ 0.0001), while those <8 (7.41 ± 0.10%, 103.45 ± 2.37 kg; *n* = 84) significantly reduced A1c by 0.38 ± 0.13 at 24 weeks (*p* = 0.0026) and body weight −5.31 ± 0.75% (*p* ≤ 0.0001). Body weight significantly decreased by 5.10 ± 0.46 kg (5.74 ± 0.50%) at week 24. The percent of participants who achieved ≥3%, ≥5%, and ≥10% weight loss at week 24 was 60%, 43%, and 15%, respectively. Participants experienced significant reductions in waist circumference (5.91 ± 0.53 cm) and diastolic blood pressure (2.8 ± 1.0 mmHg) at week 24. At week 24, there were significant reductions in overall DDS score (0.33 ± 0.10) as well as the Emotional Burden (0.33 ± 0.12) and Regimen Related Distress (0.62 ± 0.13) subscales, respectively (Table 2). Further, there were significant reductions in the IWQOL-L overall and in all subscales at week 24. Lastly, at week 24, hunger was significantly reduced (14 ± 2).

**Table 1:** Change in hemoglobin A1c, body weight, waist circumference, and blood pressure with a 24 week scalable, virtually delivered diabetes-tailored weight management program.

Parameter	Baseline	∆ Week 12	*P*-Value	∆ Week 24	*P*-Value
HbA1c	8.00 ± 0.09	−0.63 ± 0.09	*p* < 0.0001	−0.75 ± 0.11	*p* < 0.0001
Weight (kg)	101.10 ± 1.47	−3.91 ±0.33	*p* < 0.0001	−5.10 ± 0.46	*p* < 0.0001
Weight (%)		−4.60 ± 0.49	*p* < 0.0001	−5.74 ± 0.50	*p* < 0.0001
Waist circumference (cm)	117.18 ± 1.08	−3.58 ± 0.38	*p* < 0.0001	−5.91 ± 0.53	*p* < 0.0001
Body mass index (kg/m^2^)	36.86 ± 0.47	−1.42 ± 0.12	*p* < 0.0001	−1.85 ± 0.16	*p* < 0.0001
Blood Pressure (mmHg)					
Systolic	129.3 ± 1.8	−4.7 ± 1.3	*p* = 0.0005	−3.1 ± 1.7	*p* = 0.0630
Diastolic	76.9 ± 0.9	−4.2 ± 0.8	*p* < 0.0001	−2.8 ± 1.0	*p* = 0.0076

Mean ± SEM.

**Table 2:** Change in diabetes distress scale, hunger, and quality of life with a 24 week scalable, virtually delivered diabetes-tailored weight management program.

DDS total score	2.35 ± 0.08	−0.23 ± 0.08	*p* = 0.0034	−0.33 ± 0.10	*p* = 0.0007
Emotional burden	2.58 ± 0.11	−0.21 ± 0.09	*p* = 0.0261	−0.33 ± 0.12	*p* = 0.0062
Regimen distress	3.10 ± 0.11	−0.51 ± 0.10	*p* < 0.0001	−0.62 ± 0.13	*p* < 0.0001
Interpersonal distress	1.92 ± 0.10	−0.11 ± 0.10	*p* = 0.2420	−0.21 ± 0.12	*p* = 0.0853
Physician distress	1.42 ± 0.08	0.03 ± 0.08	*p* = 0.6926	−0.04 ± 0.10	*p* = 0.6583
Hunger	55 ± 2	−15 ± 2	*p* < 0.0001	−14 ± 2	*p* < 0.0001
IWQOL – Lite (t)	72 ± 2	6 ± 1	*p* < 0.0001	9 ± 1	*p* < 0.0001
Physical function	69 ± 2	7 ± 1	*p* < 0.0001	10 ± 1	*p* < 0.0001
Self – esteem	59 ± 2	8 ± 1	*p* < 0.0001	12 ± 2	*p* < 0.0001
Sexual life	75 ± 3	6 ± 2	*p* = 0.0035	8 ± 3	*p* = 0.0014
Public distress	84 ± 2	2 ± 1	*p* = 0.0371	6 ± 1	*p* < 0.0001
Work	82 ± 2	5 ± 2	*p* = 0.0008	6 ± 2	*p* = 0.0047

Mean ± SEM.

*DDS* Diabetes Distress Scale, *IWQOL* impact of weight on quality of life.

## Discussion

The WW virtual weight loss and wellness program tailored for diabetes resulted in HbA1c reductions and improvements in diabetes distress similar to in-person trials [5, 18]. Of note, the >0.5% reduction in HbA1c, >5% weight loss, and decrease in DDS to from moderate to low levels of diabetes-related distress are all clinically meaningful [19–21]. The observed HbA1c reduction compares favorably to in-person community-based approaches, two of which included portion controlled meals [22, 23] as well as an earlier version of WW that included at least 2 individual sessions with a certified diabetes care and education specialist [11]. A recent meta-analysis found that more intensive interventions promoted reductions in HbA1c and DDS to those found in the current trial [24]. Another area of potential future study may be to evaluate the programming in comparison or addition to accredited diabetes self-management education/training (DSME/T).

### Limitations

These promising results await replication in a randomized controlled trial. While it is possible that the observed effects were simply a regression to the mean, the baseline A1c of <8 suggest this is less likely. It is also unlikely that a nearly 6% weight loss occurs spontaneously in the absence of a structured weight management program.

## Conclusion

A T2D-tailored virtual, widely available weight management program produced favorable change in glycemic control, weight, diabetes distress, quality of life, and CVD risk factors among people living with T2D and overweight/obesity. Moreover, since this program has been shown to be a cost-effective weight loss program [8] and is delivered via a digital platform, it has the potential to mitigate access and affordability barriers for adults with T2D seeking weight management to improve their glycemic control and other CVD risk factors.
